# Dietary inclusion of defatted black soldier fly larvae meal: impacts on laying hen performance, egg quality, serum biomarkers, and intestinal morphology

**DOI:** 10.3389/fvets.2025.1605077

**Published:** 2025-06-13

**Authors:** Lifei Chen, Haoyang Sun, Hanhan Song, Guiying Wang, Xiuliang Ma, Jiacai Tu, Lei Yang, Junxia Li, Yuxi Wang, Xueqiang Meng, Wenyu Zhang, Shenghao Li, Qile Tian, Yinling Zhao, Hongyan Yang, Peixian Wang, Lusheng Li

**Affiliations:** ^1^College of Agriculture and Biology, Shandong Province Engineering Research Center of Black Soldier Fly Breeding and Organic Waste Conversion, Liaocheng University, Liaocheng, China; ^2^Shandong Fengxiang Co., Ltd., Liaocheng, China; ^3^Liaocheng City Agricultural Technology Extension Service Center, Liaocheng, China; ^4^Yantai Tianhua Breeding Co., Ltd., Yantai, China

**Keywords:** defatted BSFL meal, egg production performance, eggshell strength, antioxidant capacity, intestinal morphology

## Abstract

This study investigated the effects of 3% (G3), 6% (G6), and 9% (G9) dietary defatted black soldier fly larvae (BSFL) meal on 288 Hy-Line Brown laying hens over 210 days. While egg production and weight showed no significant differences (*p* > 0.05), feed-to-egg ratios increased in higher inclusion groups (G6, G9) versus 0% control (G0) during later phases (*p* < 0.01). G6 exhibited enhanced eggshell strength versus G0 (*p* < 0.05), while G3 demonstrated thicker eggshells than all groups (*p* < 0.05). Serum analysis revealed G3 had superior total antioxidant capacity and lower lipid peroxidation versus G0 and G9 (*p* < 0.05), along with elevated gonadotropin-releasing hormone levels compared to G9 (*p* < 0.05). Intestinal morphology remained unaffected across treatments. The 3% BSFL inclusion optimally balanced eggshell quality and antioxidant status under isoenergetic-isoprotein conditions, supporting its viability as a sustainable protein source in poultry diets. Findings advocate BSFL meal as an eco-friendly feed alternative, with 3% identified as the most effective inclusion rate.

## 1 Introduction

The growth of the global population and the development of the economy have driven the expansion of the livestock industry. However, the shortage of protein feed, especially soybean meal and fish meal, has become a bottleneck restricting the development of animal husbandry. There is an urgent need to find alternative protein resources. Black soldier fly larvae (BSFL) are considered a highly promising protein feed resource that can help alleviate the shortage of protein resources in livestock development (1–4). Currently, the use of BSFL in layer chicken feed is mostly based on proportionally replacing soybean meal or fish meal (5–7). Some studies have used energy conversion methods, but the energy data used are gross energy rather than the commonly used metabolizable energy. A few studies have directly added live larvae to the feed (8, 9). Due to the different methods of use, the effects vary greatly (10). Some studies have shown that replacing 15% of soybean meal with BSFL meal has no adverse effects on layer chicken production performance, and there are even reports that complete replacement of soybean meal with BSFL meal does not reduce layer chicken performance (6, 11, 12). However, Khan et al. (13) found that adding 9% BSFL meal does not affect layer chicken production performance, but an addition level of 18% significantly reduces egg production. This suggests that excessive BSFL meal can affect layer chicken egg production. The majority of studies have reported similar results. The reason is analyzed to be related to the different nutritional values of BSFL meal and the presence of the anti-nutritional factor chitin (11, 14, 15). The nutritional value of BSFL meal varies greatly depending on its source, resulting in different levels of metabolizable energy, protein, and essential amino acids in the experimental diets, which in turn leads to differences in the results (16).

Poultry egg-laying behavior is closely related to energy and protein (17, 18). Both insufficient and excessive energy can affect the expression of production performance. The amino acids in eggs depend on the supply of dietary amino acids, and adequate dietary amino acids are crucial for maintaining poultry production efficiency (19). There are differences in the requirements for metabolizable energy, protein, and amino acids in different egg-laying stages of layer chickens. In the early and peak stages, higher levels of metabolizable energy and protein are needed due to the higher production performance. As egg-laying capacity declines in the later stages, the levels of metabolizable energy and protein in the diet should be appropriately reduced (20). Current research on the application of BSFL meal in layer chickens mostly focuses on the late egg-laying period (13, 21, 22), and a few studies have focused on the peak egg-laying period (10, 23). So far, there have been no reports on the application of BSFL meal throughout the entire egg-laying period.

This study addresses the above issues by using the metabolizable energy and nutrient utilization data determined by our research group (24). Based on the principle of isocaloric and isoprotein diets, experimental diets with different proportions of BSFL meal were formulated. Using the initial stage of the peak-laying hens as the research subject, the study investigated the effects of BSFL meal on egg production performance, egg quality, blood biochemical indices, antioxidant capacity, reproductive hormones, intestinal mucosal structure, and intestinal microbiota in layer chickens from 30 to 60 weeks of age. This research aims to provide theoretical basis and data support for the application of BSFL meal in layer chicken diets in practical production.

## 2 Materials and methods

### 2.1 Preparation of defatted BSFL meal

BSFL eggs were sourced from Shandong Wo Neng Agricultural Technology Co., Ltd. (Liaocheng city, China). After hatching at 32°C, the larvae were reared for 3 days on a substrate composed of bran, soybean meal, and cornmeal, and then transferred to kitchen waste slurry (sourced from Liaocheng University Boyuan Restaurant) for an additional 10 days of rearing (3). The larvae were then separated, washed, and dried by heating. Following the method of Kong et al. (25), the dried larvae were placed in an extraction vessel with hexane at a ratio of 1:5 (larvae to hexane) and extracted at 60°C for 5 h. The larvae were subsequently air-dried after oil extraction and ground into defatted BSFL meal.

### 2.2 Selection of experimental animal

The present study was conducted in accordance with the methods approved by the Animal Care and Use Committee of Liaocheng University, China (AP2024022927). The experimental animals were provided by Shandong Wo Neng Agricultural Technology Co., Ltd. (Liaocheng city, China). A total of 288 healthy 29-week-old Hy-Line Brown laying hens (W-36) with similar body weight, consistent feeding management, similar age at onset of lay, and comparable egg production performance were selected. The hens were randomly divided into four treatment groups (G0, G3, G6, G9) based on similar egg production rate and body weight. Each group consisted of six replicates, with 12 hens per replicate. The hens were housed in a three-tier cage system, with each replicate occupying one cage on the upper, middle, and lower levels (dimensions: 47 cm × 37 cm × 43 cm).

### 2.3 Experimental diet formulation

The experimental diets were based on a corn-soybean meal type, formulated according to the nutritional requirements of GB/T 5916–2020 “Compound Feed for Laying and Meat Chickens” and the 2018 “Hy-Line Brown Laying Hen Breeding Manual” for pullets. The defatted BSFL meal was prepared using the method described in Xin et al. (24). The metabolizable energy and nutrient digestibility data of defatted black soldier fly larvae meal were obtained from our research group’s findings (26). Other nutrient levels were designed with reference to the “Chicken Feeding Standards” (NY/T33-2004) and the “China Feed Composition and Nutritional Value Table (2018, 29th Edition).” The composition and nutritional levels of the experimental diets are shown in Table 1. Group G0 was the control group, fed a basal diet without BSFL meal. Groups G3, G6, and G9 were experimental groups, fed diets supplemented with 3, 6, and 9% BSFL meal, respectively.

**Table 1:** Composition and nutrient levels of experimental diets.

Items (%)	Inclusion levels^1^, %			
	G0	G3	G6	G9
Ingredients (air-dry basis)				
Corn	60.46	60.44	59.65	58.98
Soybean meal	22.55	19.24	16.17	14.05
Corn gluten meal	2.00	2.07	3.25	2.50
Bran	2.50	3	3.05	3.00
Defatted BSFL meal	0.00	3	6	9.00
Soybean oil	1.50	1.5	1.5	1.50
Limestone	8.13	8.08	7.96	8.75
CaHPO_4_	1.36	1.2	1.03	0.87
Salt	0.38	0.23	0.1	0.00
L-Lys·HCl (98%)	0	0.1	0.13	0.17
DL-Met	0.12	0.14	0.16	0.18
Premix	1.00	1	1	1.00
Total	100.00	100.00	100.00	100.00
Nutrient levels (dry matter basis)				
Crude protein, %	16.70	16.71	16.71	16.72
Metabolizable energy, MJ/kg	11.51	11.51	11.52	11.51
Calcium, %	3.45	3.51	3.51	3.52
Non-phytate phosphorus, %	0.41	0.41	0.42	0.42
NaCl, %	0.36	0.36	0.37	0.36
Lysine, %	0.81	0.83	0.82	0.82
Methionine, %	0.38	0.39	0.39	0.38

*The crude protein, lysine, and methionine levels were measured values; other nutrient levels were calculated values. BSFL, black soldier fly larvae.

1 G0, diet supplemented with 0% BSFL meal; G3, diet supplemented with 3% BSFL meal; G6, diet supplemented with 6% BSFL meal; G9, diet supplemented with 9% BSFL meal.

### 2.4 Feeding management

The experiments were carried out in the agricultural ecological park of Liaocheng, University. The experimental period consisted of a 2-week transition phase followed by a 210-day formal trial, divided into three stages: 31–40 weeks, 41–50 weeks, and 51–60 weeks. The hens were fed twice daily at 07:00 and 15:00, with free access to water via nipple drinkers. The lighting was maintained at a constant 16 h per day, and natural ventilation and clean housing conditions were ensured. Routine vaccination and immunization were carried out during the trial period.

### 2.5 Measurement of egg production performance

The feed intake, number of eggs laid, and daily egg weight of each replicate were recorded weekly. The egg production rate, average egg weight, average daily feed intake, and feed-to-egg (F/E) ratio were calculated for each stage.

### 2.6 Measurement of egg quality

On the last day of the 50th week, two eggs were randomly selected from each replicate and analyzed using an egg quality analyzer (Egg Shell Strength Meter II, Fujihira, Tokyo, Japan). The measured indicators included egg shape index, eggshell strength and thickness, albumen height, yolk color, and Haugh unit. The yolk was separated using a yolk sampler, and the yolk weight was measured using an analytical balance. The yolk percentage was calculated using the following formula:

Yolk percentage=Yolk weight/Eggweight×100%.

### 2.7 Blood sample collection and analysis

On the last day of the 50th week, two hens were randomly selected from each replicate, so a total of 12 hens were selected from each group. Blood was collected via the wing vein (4 milliliters of blood was collected from each hen). The blood samples were centrifuged at 4000 r/min for 15 min to separate the serum, which was stored at −80°C for subsequent analysis (24).

Serum biochemical indices, including alanine aminotransferase (ALT), aspartate aminotransferase (AST), total protein (TP), albumin (ALB), globulin (GLO), blood urea nitrogen (BUN), uric acid (UA), cholesterol (TC), triglycerides (TG), and glucose (GLU), were measured using an automatic biochemical analyzer (Cobas-8000 c702, Basel, Switzerland). Antioxidant indices, including total antioxidant capacity (T-AOC), glutathione peroxidase (GSH-Px), malondialdehyde (MDA), and total superoxide dismutase (T-SOD), as well as reproductive hormones, including luteinizing hormone (LH), follicle-stimulating hormone (FSH), estradiol (E2), progesterone (P4), and gonadotropin-releasing hormone (GnRH), were measured by the Animal Nutrition and Feed Analysis Laboratory of Liaocheng University using reagent kits from Nanjing Jiancheng Bioengineering Institute (Nanjing, China), following the manufacturer’s instructions.

### 2.8 Intestinal health indicators

On the last day of the 60th week, one hen was randomly selected from each replicate and slaughtered by jugular vein exsanguination after a 12-h fast. The jejunum and ileum were quickly removed, and approximately 2 cm of intestinal segments were excised, washed carefully with 0.9% saline, blotted dry with filter paper, and fixed in 4% paraformaldehyde solution. Intestinal sections were prepared according to the method of Marta et al. (27) to measure villus height and crypt depth, and the villus height-to-crypt depth ratio (VH/CD) was calculated.

### 2.9 Statistical methods

The experimental data were organized using Excel 2019 and the performance, egg quality, serum analysis, and intestinal measurements were analyzed using a one-way ANOVA through SPSS 19.0 software (version 22.0, SPSS for Windows, release 19.0, developed by SPSS Inc., Chicago, IL, USA). Shapiro–wilk test was applied for normal distribution check. Levene’s test was used for homogeneity of variance test. Duncan’s multiple range test was used to compare the differences among group means, with significance set at *p* < 0.05. The results are presented as mean ± standard deviation (mean ± SD).

## 3 Results

### 3.1 Effects of defatted BSFL meal on egg production performance of 31–40 week-old laying hens

The effects of defatted BSFL meal on the laying performance of laying hens are shown in Table 2. The results showed that during the three stages (31–40 weeks, 41–50 weeks, and 51–60 weeks), the egg production rate and average egg weight of the three treatment groups (G3, G6, and G9) were not significantly different from those of the control group (G0) (*p* > 0.05). In terms of feed intake, the average daily feed intake of the G6 group was significantly higher than that of the G0 group in all three stages (*p* < 0.05), and the G9 group also showed significantly higher feed intake during the 41–50 week and 51–60 week stages (*p* < 0.05). However, the G3 group showed no significant difference in feed intake compared to the G0 group (*p* > 0.05). Regarding the feed-to-egg ratio, the G6 group had a significantly higher ratio than the G0 and G3 groups during the 31–40 week stage (*p* < 0.05) and extremely significantly higher during the 41–50 week and 51–60 week stages (*p* < 0.01). The G9 group also had an extremely significantly higher feed-to-egg ratio than the G0 group during the 41–50 week and 51–60 week stages (*p* < 0.01), with significant differences compared to the G3 group (*p* < 0.05). Additionally, the feed-to-egg ratio of the G3 group was significantly higher than that of the G0 group during the 41–50 week and 51–60 week stages (*p* < 0.05), but no significant difference was observed during the 31–40 week stage (*p* > 0.05).

**Table 2:** Effects of skimmed BSFL meal on egg-laying performance of layers.

Items	Inclusion levels^1^			
	G0	G3	G6	G9
31–40 week-old				
Laying rate, %	96.23 ± 3.11	96.51 ± 2.44	96.12 ± 3.01	96.02 ± 2.61
Average egg weight, g	59.39 ± 1.39	59.46 ± 1.15	59.24 ± 1.46	59.24 ± 1.30
ADFI, g/d	120.30 ± 2.84a	121.49 ± 1.36a	123.60 ± 3.03b	121.45 ± 2.20a
F/E	2.03 ± 0.01a	2.04 ± 0.05a	2.09 ± 0.01b	2.05 ± 0.03ab
41–50 week-old				
Laying rate, %	92.54 ± 3.01	92.59 ± 2.07	91.99 ± 2.44	91.33 ± 2.37
Average egg weight, g	60.37 ± 1.20	60.51 ± 1.30	59.86 ± 1.46	60.02 ± 1.29
ADFI, g/d	120.44 ± 2.36a	122.68 ± 2.35ab	126.67 ± 2.77b	123.83 ± 2.45b
F/E	2.00 ± 0.01Aa	2.03 ± 0.01AbC	2.12 ± 0.01B	2.06 ± 0.01Cc
51–60 week-old				
Laying rate, %	89.70 ± 2.82	89.75 ± 2.50	89.15 ± 2.70	88.49 ± 1.82
Average egg weight, g	61.38 ± 1.20	61.52 ± 1.30	60.87 ± 1.46	61.03 ± 1.29
ADFI, g/d	120.72 ± 2.29a	122.96 ± 2.56ab	126.95 ± 2.77b	124.11 ± 2.45b
F/E	1.97 ± 0.00Aa	2.00 ± 0.01AbC	2.09 ± 0.01B	2.03 ± 0.01Cc

1 G0, diet supplemented with 0% BSFL meal; G3, diet supplemented with 3% BSFL meal; G6, diet supplemented with 6% BSFL meal; G9, diet supplemented with 9% BSFL meal.

ADFI, average daily feed intake; F/E, feed-to-egg. In the table, lower case letters indicate significant differences (*p* < 0.05), upper case letters indicate extremely significant differences (*p* < 0.01), and the same letters or no labels indicate no significant differences at the 0.05 level within a row. Values are mean ± SEM (*n* = 6).

### 3.2 Effects of defatted BSFL meal on egg quality at 50 weeks of age

The effects of defatted BSFL meal on egg quality of 50-week-old laying hens are shown in Table 3. The results indicated that compared to the G0 group, the G3, G6, and G9 groups showed no significant differences in egg shape index, albumen height, Haugh unit, yolk color, and yolk percentage (*p* > 0.05). The G6 group had significantly higher eggshell strength than the G0 group (*p* < 0.05), but no significant differences were observed between the G6 group and the G3 or G9 groups (*p* > 0.05). The G3 group had significantly higher eggshell thickness compared to the G0, G6, and G9 groups (*p* < 0.05), while no significant differences were found among the G0, G6, and G9 groups (*p* > 0.05).

**Table 3:** Effects of skim BSFL meal on egg quality of 50 week-old eggs.

Items	Inclusion levels^1^			
	G0	G3	G6	G9
Egg shape index	1.34 ± 0.03	1.35 ± 0.03	1.36 ± 0.02	1.35 ± 0.04
Eggshell strength, kg/cm^2^	3.95 ± 0.24a	4.21 ± 0.19ab	4.26 ± 0.28b	4.16 ± 0.07ab
Eggshell thickness, mm	0.39 ± 0.02a	0.41 ± 0.02b	0.39 ± 0.02a	0.38 ± 0.01a
Albumen height, mm	5.27 ± 0.36	5.48 ± 0.35	5.46 ± 0.48	5.33 ± 0.45
Haugh unit	74.55 ± 9.37	80.12 ± 7.66	77.95 ± 10.69	80.59 ± 6.96
Egg yolk color	4.22 ± 0.16	4.29 ± 0.18	4.28 ± 0.20	4.18 ± 0.17
Egg yolk ratio, %	28.75 ± 2.72	30.27 ± 2.72	29.28 ± 1.83	28.86 ± 0.97

1 G0, diet supplemented with 0% BSFL meal; G3, diet supplemented with 3% BSFL meal; G6, diet supplemented with 6% BSFL meal; G9, diet supplemented with 9% BSFL meal.

In the table, lower case letters indicate significant differences (*p* < 0.05), and the same letters or no labels indicate no significant differences at the 0.05 level within a row. Values are mean ± SEM (*n* = 6).

### 3.3 Effects of defatted BSFL meal on biochemical indices at 50 weeks of age

The effects of defatted BSFL meal on the biochemical indicators of 50-week-old laying hens are shown in Table 4. The results showed that compared to the G0 group, there were no significant differences in cholesterol, triglycerides, glucose, ALT, and AST among the three treatment groups (G3, G6, and G9) (*p* > 0.05). Additionally, no significant differences were observed among the different addition levels of defatted BSFL meal (*p* > 0.05).

**Table 4:** Effects of skim BSFL meal on biochemical indexes of laying hens aged 50 weeks.

Items	Inclusion levels^1^			
	G0	G3	G6	G9
TP, g/L	49.16 ± 2.03	47.54 ± 2.21	48.42 ± 2.83	48.41 ± 2.82
ALB, g/L	16.88 ± 1.66	17.12 ± 0.68	16.72 ± 1.48	16.82 ± 0.95
GLO, g/L	32.28 ± 3.36	30.43 ± 1.77	31.70 ± 2.58	31.59 ± 2.61
UN, mmol/L	0.30 ± 0.02	0.29 ± 0.01	0.31 ± 0.01	0.30 ± 0.02
TC, mmol/L	3.84 ± 0.30	3.81 ± 0.31	3.86 ± 0.30	3.73 ± 0.28
TG, mmol/L	0.44 ± 0.05	0.45 ± 0.08	0.43 ± 0.08	0.43 ± 0.06
GLU, mmol/L	15.19 ± 0.48	15.38 ± 0.58	14.88 ± 0.59	14.86 ± 0.16
ALT, U/L	24.15 ± 1.13	24.34 ± 1.05	24.36 ± 0.27	25.61 ± 2.83
AST, U/L	168.56 ± 4.27	175.78 ± 6.52	171.98 ± 3.97	172.87 ± 5.49

TP, total serum protein; ALB, albumin; GLOB, globulin; CHOL, cholesterol; TG, triglyceride; UN, urea nitrogen; GLU, glucose; ALP, alkaline phosphatase; ALT, alanine aminotransferase; AST, aspartate aminotransferase.

1 G0, diet supplemented with 0% BSFL meal; G3, diet supplemented with 3% BSFL meal; G6, diet supplemented with 6% BSFL meal; G9, diet supplemented with 9% BSFL meal. Values are mean ± SEM (*n* = 6).

### 3.4 Effects of defatted BSFL meal on antioxidant indices and reproductive hormones at 50 weeks of age

The results of the effects of defatted BSFL meal on antioxidant indices and reproductive hormones of 50-week-old laying hens are shown in Table 5. The results indicated that the G3 group had significantly higher T-AOC compared to the G0 and G9 groups (*p* < 0.05), while no significant differences were found among the G0, G6, and G9 groups (*p* > 0.05). The G6 group had significantly higher GSH-Px than the G0 group (*p* < 0.05), with no significant differences among the G0, G3, and G9 groups (*p* > 0.05). The G3 group had significantly lower MDA compared to the G0 and G9 groups (*p* < 0.05), while no significant differences were observed among the G0, G6, and G9 groups (*p* > 0.05). No significant differences were found in T-SOD among the four treatment groups (*p* > 0.05). Regarding reproductive hormones, the G3 group had significantly higher GnRH compared to the G9 group (*p* < 0.05), with no significant differences between the G3 group and the G0 or G6 groups (*p* > 0.05). No significant differences were observed among the four treatment groups in terms of LH, FSH, E2, and P4 (*p* > 0.05).

**Table 5:** Effects of skim BSFL meal on antioxidant indexes and reproductive hormones in 50-week-old laying hens.

Items	Inclusion levels^1^			
	G0	G3	G6	G9
T-AOC, U/mL	8.74 ± 0.43a	9.40 ± 0.22b	9.14 ± 0.23ab	8.90 ± 0.33a
GSH-Px, U/mL	454.39 ± 5.56a	461.57 ± 4.85ab	467.74 ± 9.28b	458.99 ± 8.72ab
MDA, nmol/mL	6.35 ± 0.10a	5.96 ± 0.40b	6.14 ± 0.25ab	6.31 ± 0.07a
T-SOD, U/mL	140.48 ± 21.55	149.94 ± 18.79	151.89 ± 5.78	137.96 ± 6.46
LH, mIU/mL	7.25 ± 0.11	7.41 ± 0.32	7.34 ± 0.23	7.27 ± 0.25
FSH, mIU/mL	7.64 ± 0.22	8.05 ± 0.37	8.00 ± 0.28	7.84 ± 0.19
E2, ng/L	83.49 ± 3.52	88.10 ± 3.41	86.26 ± 3.72	84.64 ± 6.46
P4, ng/mL	16.52 ± 0.70	17.07 ± 0.68	16.76 ± 0.65	16.20 ± 0.86
GnRH, ng/L	92.99 ± 10.46ab	105.67 ± 4.21a	102.43 ± 5.63ab	90.71 ± 6.35b

T-AOC, total antioxidant capacity; GSHPx, glutathione peroxidase; MDA, malonaldehyde; T-SOD, total superoxide dismutase; LH, luteinizing hormone; FSH, follicle-stimulating hormone; E2, estradiol; P4, progesterone; GnRH, gonadotropin-releasing hormone.

1 G0, diet supplemented with 0% BSFL meal; G3, diet supplemented with 3% BSFL meal; G6, diet supplemented with 6% BSFL meal; G9, diet supplemented with 9% BSFL meal. Note: In the table, lower case letters indicate significant differences (*p* < 0.05), and the same letters or no labels indicate no significant differences at the 0.05 level within a row. Values are mean ± SEM (*n* = 6).

### 3.5 Effects of defatted BSFL meal on intestinal morphology at 60 weeks of age

**Figure 1:**
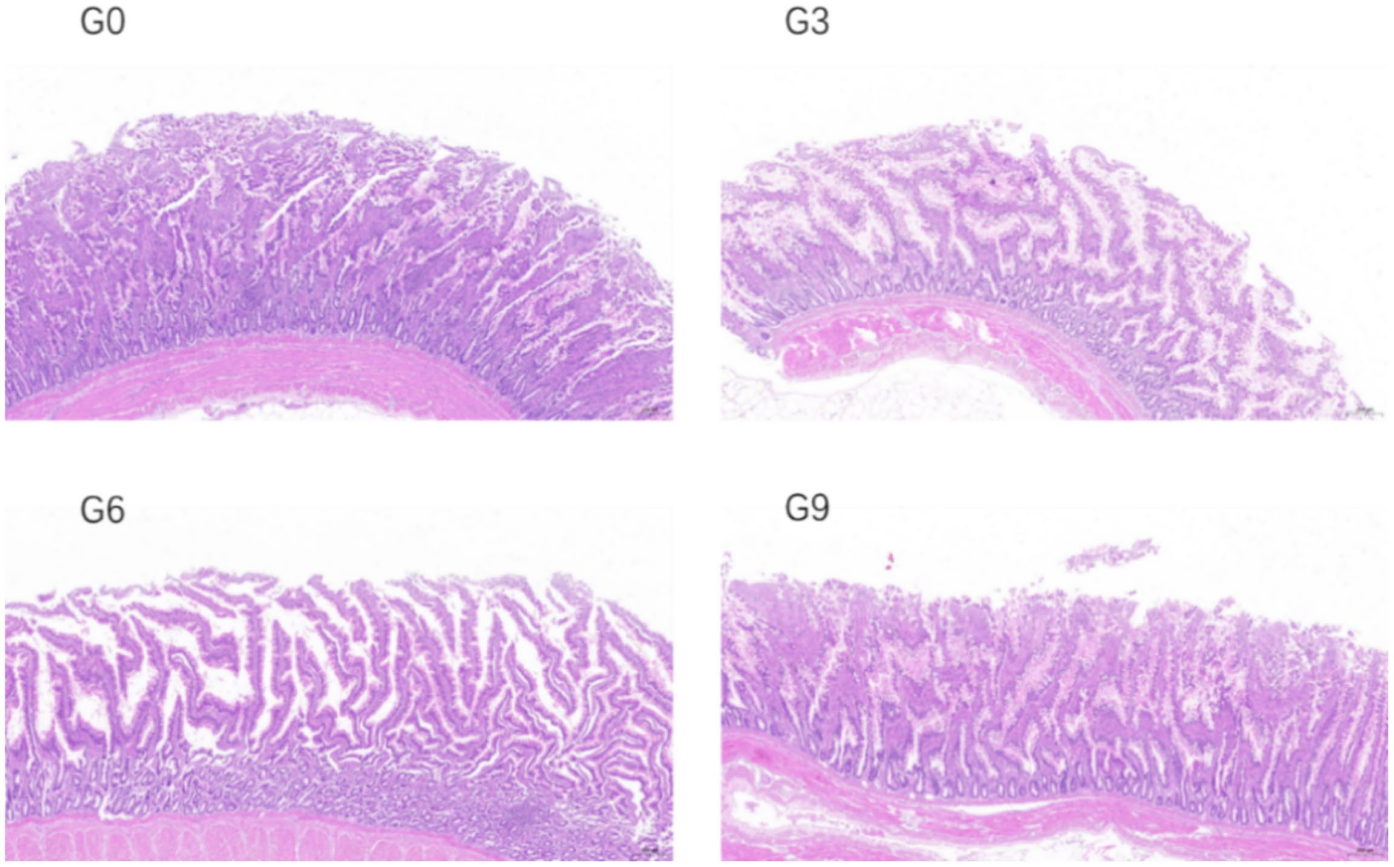
Effect of skim defatted BSFL meal on the structure of jejunum mucosa in laying hens (40 times).

**Figure 2:**
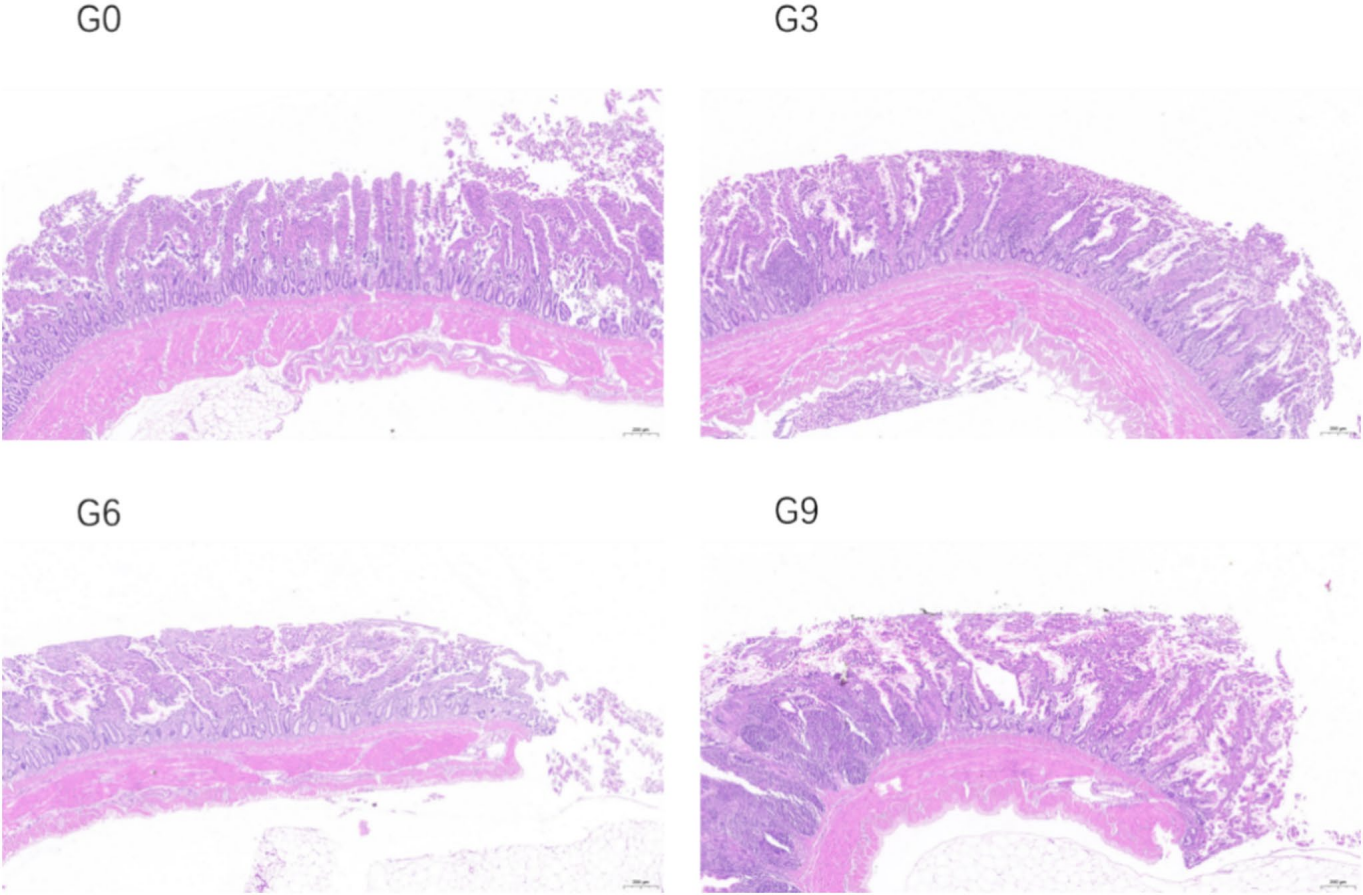
Effect of skim defatted BSFL meal on the structure of ileal mucosa in laying hens (40 times).

The effects of defatted BSFL meal on the morphological structure of the jejunal and ileal mucosa of laying hens are shown in Table 6. The changes in the jejunal and ileal mucosa of laying hens in different experimental groups are shown in Figures 1, 2 respectively. The results showed that compared to the G0 group, there were no significant differences in villus height, crypt depth, or VH/CD in the jejunum and ileum among the G3, G6, and G9 groups (*p* > 0.05). Additionally, no significant differences were observed among the different addition levels of defatted BSFL meal in terms of these intestinal morphology indicators (*p* > 0.05).

**Table 6:** Effects of defatted BSFL meal on the morphology and structure of jejunum and ileum in 60-week-old laying hens.

Items	Inclusion levels^1^			
	G0	G3	G6	G9
Jejunum				
Villus height, mm	0.89 ± 0.18	0.74 ± 0.09	0.89 ± 0.10	0.85 ± 0.11
Crypt depth, mm	0.15 ± 0.01	0.16 ± 0.02	0.17 ± 0.03	0.15 ± 0.04
Villus height/crypt depth	6.23 ± 1.55	4.66 ± 0.43	5.38 ± 0.84	5.77 ± 0.85
Ileum				
Villus height, mm	0.66 ± 0.18	0.58 ± 0.09	0.70 ± 0.13	0.68 ± 0.09
Crypt depth, mm	0.14 ± 0.04	0.15 ± 0.02	0.18 ± 0.04	0.15 ± 0.02
Villus height/crypt depth	5.18 ± 1.89	3.90 ± 0.39	4.19 ± 1.29	4.64 ± 1.33

1 G0, diet supplemented with 0% BSFL meal; G3, diet supplemented with 3% BSFL meal; G6, diet supplemented with 6% BSFL meal; G9, diet supplemented with 9% BSFL meal.

In the table, and the same letters or no labels indicate no significant differences at the 0.05 level within a row. Values are mean ± SEM (*n* = 6).

## 4 Discussion

This study found that during the entire laying period from peak production to later stages (31–60 weeks), the addition of 3–9% defatted BSFL meal in the diet had no significant negative impact on egg production rate and average egg weight. This finding is consistent with the results of Khan et al. (13). However, Mwaniki et al. (12) reported that completely replacing soybean meal with BSFL meal had no effect on the egg production rate of laying hens (28–43 weeks old), but average egg weight decreased linearly. Most studies have shown that BSFL meal exhibits a threshold effect in poultry feed, where low doses do not affect production performance, but high doses can lead to decreased egg production and egg weight (11, 14, 15). However, this threshold effect was not observed in the present study, possibly due to differences in the methods of using BSFL meal. Currently, the use of BSFL meal in layer chicken feed mostly involves proportionally replacing soybean meal or fish meal (5, 7). Some studies use gross energy instead of metabolizable energy, or directly add live larvae (9). These differences lead to significant variations in metabolizable energy, protein, and essential amino acids in the experimental diets, thereby affecting egg production performance. Additionally, the linoleic acid content in the experimental diets is also an important factor influencing egg weight (12). In this study, the metabolizable energy value and nutrient digestibility of BSFL meal were accurately determined, and the experimental diets were formulated based on the isocaloric and isoprotein principle, thus avoiding negative impacts on production performance.

Regarding feed intake and feed-to-egg ratio, the present study found that the average daily feed intake of the 6% addition group was significantly higher than that of the control group during the 31–40 week stage, and also significantly increased during the 41–50 week and 51–60 week stages. However, this increase in feed intake did not translate into improved egg production performance but instead led to a significant increase in the feed-to-egg ratio. Kroeckel et al. (28) reported that a high proportion (15%) of BSFL meal increased feed intake and slightly raised the feed-to-egg ratio, which is consistent with the results of this study. In contrast, Khan et al. (13) observed that adding 18% BSFL meal decreased feed intake, although the feed-to-egg ratio showed no significant difference. Most studies have shown that low doses of BSFL meal do not affect poultry production performance, but high doses can lead to decreased performance (11, 15). This may be related to the chitin content in BSFL meal. Chitin, a major component of the larvae’s exoskeleton, has immune-regulating, antibacterial, and anti-inflammatory functions (29). However, as a large molecule, it can encapsulate nutrients and hinder the action of digestive enzymes, thereby reducing feed utilization. Moreover, the degree of defatting can also affect feed intake. This study found that the fat content and metabolizable energy of larvae meal obtained by different defatting methods varied significantly, consistent with the results of Cullere et al. (30). Romero et al. (10) pointed out that the fatty acid composition of BSFL meal (e.g., lauric acid content) may influence the energy metabolism pathways in poultry, and high addition levels may cause energy allocation imbalances.

The results of this study showed that the addition of defatted BSFL meal had no significant effect on albumen height, Haugh unit, yolk color, or yolk percentage, but it could improve eggshell strength and thickness at different rearing stages. In particular, the 3 and 6% addition groups showed significant differences, while the 9% addition group, although not significant, also exhibited an upward trend. These results are consistent with those of Khan et al. (13) and Mwaniki et al. (12), but differ from those of Seolhwa et al. (31). The differences may be related to the calcium content in the diet, the properties of defatted BSFL meal, and the rearing methods. The rearing scheme in this study used caged housing, where the hens’ nutrient sources were entirely dependent on the diet. In contrast, Seolhwa et al. (31) used a free-range rearing model, where the hens could freely obtain calcium, thus no changes in eggshell thickness were observed. Some reports suggest that the fermentation of chitin in defatted BSFL meal can increase calcium absorption, thereby enhancing eggshell thickness (32). Lokaewmanee et al. (9) found that adding BSFL meal decreased yolk color and speculated that this was due to the lower content of red carotenoids in the feed. The results of this study showed no effect on yolk color, which may be related to the fat content of the BSFL meal. The larvae meal used in this study was defatted by extraction, while Seolhwa et al. (31) and Lokaewmanee et al. (9) used full-fat BSFL meal. The differences in metabolizable energy may have affected the absorption of lutein.

This study found that the addition of defatted BSFL meal had no significant effect on any of the biochemical indices in laying hens, which is consistent with the results of Khan et al. (13) and Li et al. (3). However, Attivi et al. (5) reported that completely replacing fish meal with BSFL meal significantly increased the concentrations of total protein, plasma cholesterol, and triglycerides in laying hens. This suggests that it can improve the utilization efficiency of protein and liver fat and enhance immune capacity. This difference may be related to the basal diet formulation and the quality of BSFL meal. In the study by Attivi et al. (5), the basal diet contained only soybean meal, and the optimization process involved adjusting the cereal components. Cereal proteins have lower digestibility, which may have affected overall protein absorption (33). Fat content and the degree of defatting are the main reasons for the differences in liver fat utilization efficiency (29). Additionally, chitin and its derivative chitosan may bind fats and hinder their absorption, thereby affecting fat metabolism (15). The results of this study on blood glucose and liver function are consistent with those of Yu Miao et al. (34). However, Xu et al. (35) reported in a finishing pig trial that the addition of BSFL meal significantly increased blood glucose levels but had no effect on ALT and AST activity. This difference may be related to the different experimental animal species.

The results of this study showed that the addition of an appropriate amount of BSFL meal significantly enhanced the T-AOC and GSH-Px activity in laying hens and reduced MDA content. In terms of addition levels, 3 and 6% were generally better than 9%. Many studies have reported results similar to those of this study. Dabbou et al. (36) found that adding BSFL meal to the diet of laying hens significantly increased T-SOD activity. Mengmeng et al. (37) reported that adding defatted BSFL meal to broiler feed significantly increased GSH-Px activity, which was positively correlated with the addition level. Kroeckel et al. (28) observed that adding 9% defatted BSFL meal significantly reduced MDA content in ducks, although GSH-Px activity showed no significant difference. Many aquaculture studies have also demonstrated that BSFL meal can enhance antioxidant capacity (38). Some researchers believe that the chitin in BSFL meal indirectly enhances antioxidant capacity through immune stimulation and anti-inflammatory effects. Yan et al. (39) explored the relationship between the antioxidant capacity of BSFL meal and *α*-tocopherol, finding that the meal contained 42.72 mg/kg of α-tocopherol, which is known to increase GSH-Px activity.

In this study, adding 3% defatted BSFL meal significantly increased the secretion of GnRH, but had no effect on FSH, LH, E2, or P4. From the perspective of addition levels, 6 and 9% did not increase the secretion of GnRH, indicating that an appropriate amount of defatted BSFL meal can improve production performance, while excessive amounts can lead to decreased performance. Xinyue et al. (40) reported that adding BSFL meal linearly increased the levels of FSH and P4 in the serum of 60-week-old laying hens. Liwen et al. (41) found that feeding BSFL meal to laying hens after forced molting increased the levels of FSH and LH in the serum. The results of this study differ from those reports. The differences may be related to the stage of the experimental animals and the nutritional levels. The laying hens used in this study were at the peak of egg production, with optimal ovarian function, ensuring normal secretion of reproductive hormones. In contrast, the laying hens used by Xinyue et al. (40) were 60-week-old hens in the late laying stage, and those used by Liwen et al. (41) were 140-week-old hens. Modern high-yielding laying hens are more prone to rapid ovarian decline due to high ovulation rates (42), and forced molting can also impair ovarian function, both of which affect the secretion of reproductive hormones. Nutritional levels are also an important factor causing different results. The secretion of reproductive hormones in laying hens is closely related to dietary nutritional levels. Moniello et al. (43) found that dietary nutritional levels are associated with changes in FSH and LH content. The BSFL meal used by Secci et al. (38) was full-fat and replaced soybean meal proportionally, while Liwen et al. (41) supplemented BSFL meal directly on the basis of the basal diet, which is different from the isocaloric and isoprotein diet used in this study.

This study found that adding defatted BSFL meal did not affect villus height, crypt depth, or the VH/CD in the jejunum and ileum. Khan et al. (13) used the isocaloric and isoprotein method to formulate diets for pullets and observed no effect of adding BSFL meal onvillus height, crypt depth, or VH/CD. Similar results were reported that a 25% replacement of soybean meal with BSFL meal did not affect villus height in the ileum of laying hens (44). These results are consistent with those of the present study. However, many studies have shown that adding BSFL meal can improve intestinal morphology. Changqing et al. (45) reported that adding full-fat BSFL meal to broiler feed significantly increased villus height in the duodenum, jejunum, and ileum. Similar results have been reported in multiple studies (46, 47). Nevertheless, Biasato et al. (44) found that compared to the soybean meal group, the group fed BSFL meal had lower villus height and V/C in the duodenum and jejunum. The regulatory effect of BSFL meal on intestinal morphology is generally believed to be related to its antibacterial and anti-inflammatory properties. The main active component in BSFL oil is lauric acid. Most studies have shown that BSFL oil can improve the intestinal morphology of livestock and poultry (48, 49). Therefore, the degree of defatting of BSFL meal can lead to differences in its regulatory effects on intestinal morphology. In this study, the larvae meal was defatted using an extraction method, resulting in low residual fat content, which may be the main reason for the lack of effect on villus height and crypt depth.

## 5 Conclusion

In summary, under the experimental conditions, formulating diets based on the principle of isocaloric and isoprotein optimization and adding defatted BSFL meal did not negatively affect egg production performance or blood biochemical indices in laying hens during the egg-laying period. However, it significantly enhanced the antioxidant capacity and levels of GnRH in the hens, while having no impact on intestinal mucosal structure. These results indicate that the use of defatted BSFL meal in the formulation of laying hen diets is feasible. From the perspective of addition levels, a 3% inclusion rate was found to be the most effective.

## Data Availability

The original contributions presented in the study are included in the article/supplementary material, further inquiries can be directed to the corresponding authors.
